# Supplementary material for: Effects of Selective Peroxisome Proliferator Activated Receptor Agonists on Corneal Epithelial Wound Healing
Source: Pharmaceuticals (Basel). 2021 Jan 25;14(2):88. doi: 10.3390/ph14020088 (PMC7911852; doi:10.3390/ph14020088)
Supplement: Supplementary file 1 [file pharmaceuticals-14-00088-s001.pdf]

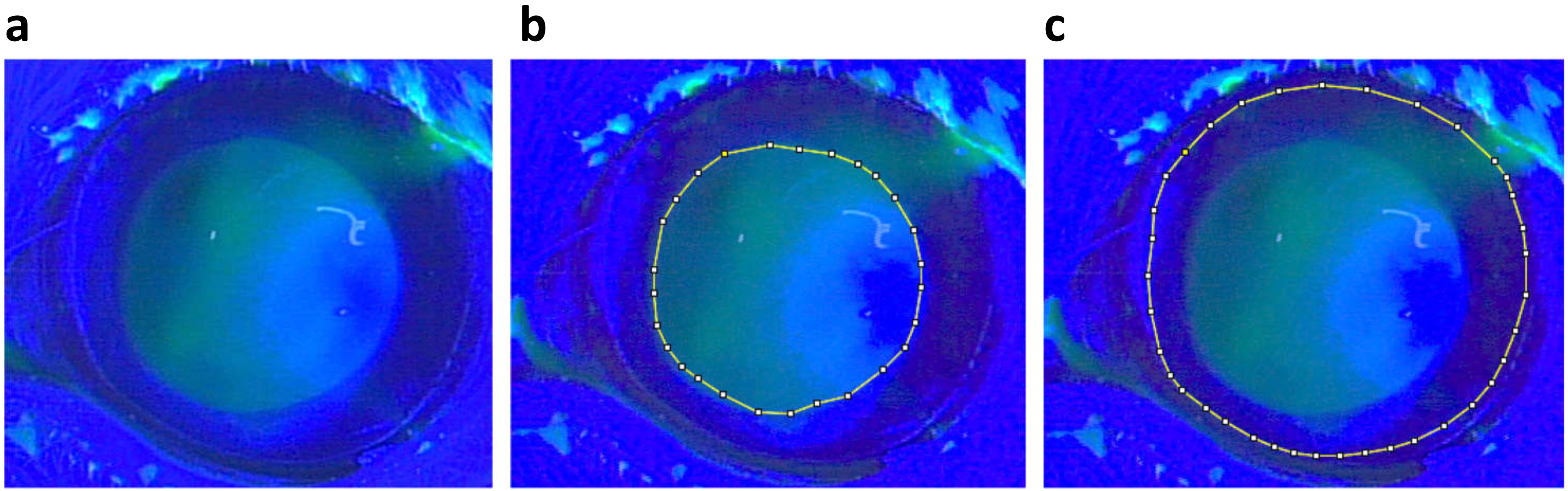

Figure 1. (**a**): Corneal epithelial defects were stained with fluorescein solution and macroscopic photographs were taken under a blue filter. Green areas were considered the corneal epithelium defect area. The area ratio of the green areas (**b**) to the entire cornea (**c**) surrounded by the yellow line was calculated using Fiji software.
